# Supplementary material for: Hanging under the ledge: synergistic consequences of UVA and UVB radiation on scyphozoan polyp reproduction and health
Source: PeerJ. 2023 Feb 2;11:e14749. doi: 10.7717/peerj.14749 (PMC9899436; doi:10.7717/peerj.14749)
Supplement: Supplemental Information 1 — Comparison of the effects of UVA and UVB on Aurelia aurita polyp weekly budding rate (buds week−1). Values in bold are significant at p < 0.05. df = degrees of freedom. [file peerj-11-14749-s001.docx]

| **Predictor** | ***df*** | **SS** | **Mean Sq** | **F-value** | ***p*-value** |
| --- | --- | --- | --- | --- | --- |
| **UVA** | 1 | 0.99 | 0.988 | 1.384 | 0.245 |
| **UVB** | 1 | 16.31 | 16.313 | 22.855 | **<0.001** |
| **UVA × UVB** | 1 | 6.13 | 6.127 | 8.585 | **0.005** |
| **Residuals** | 50 | 35.69 | 0.714 |  |  |
