# Supplementary material for: Hanging under the ledge: synergistic consequences of UVA and UVB radiation on scyphozoan polyp reproduction and health
Source: PeerJ. 2023 Feb 2;11:e14749. doi: 10.7717/peerj.14749 (PMC9899436; doi:10.7717/peerj.14749)
Supplement: Supplemental Information 2 — Comparison of Aurelia aurita polyp weekly budding rate (buds week−1) across UVR treatments. AA = UVA only; BB = UVB only; AB = UVA + UVB. Values in bold are significant at p < 0.05. [file peerj-11-14749-s002.docx]

| **Pairwise comparisons** | **Difference** | **95% Lower CI** | **95% Upper CI** | ***p*-value** |
| --- | --- | --- | --- | --- |
| **AA vs Control** | 0.46 | -0.38 | 1.30 | 0.467 |
| **BB vs Control** | -0.49 | -1.33 | 0.35 | 0.413 |
| **AB vs Control** | -1.40 | -2.23 | -0.56 | **<0.001** |
| **BB vs AA** | -0.95 | -1.87 | -0.0.3 | **0.039** |
| **AB vs AA** | -1.86 | -2.77 | -0.94 | **<0.001** |
| **AB vs BB** | -0.91 | -1.82 | 0.01 | 0.053 |
